# Supplementary material for: Chromothripsis during telomere crisis is independent of NHEJ, and consistent with a replicative origin
Source: Genome Res. 2019 May;29(5):737–49. doi: 10.1101/gr.240705.118 (PMC6499312; doi:10.1101/gr.240705.118)
Supplement: Supplemental Material [file supp_gr.240705.118_Supplemental_file_1.zip › contigs/annotated_contigs/DB116/contig.4.DB116_length_864_mean_cov_6.09259259259.docx]

**DB116_length_864_mean_cov_6.09259259259**

```
TGTGAGAGAGATGTGGGATTGAATTATGCAATGTATATATCACACTTTACACAGTATCCAACACAGAAATAAATGCCAGATTCATGGTT
 >chr2:49445134-49445366 - E=1e-124
GTTGTTTTTTTTCATCTCCCTCTAGAGTGTCTTCTGCCCTCTAATGACCTACCTGCCCCATGAGCCTCACTCTTCATTGCCTAGGGTAG

GTAATCAGCTTCCTTCCCCTCACCTTCTGCCATCTGTCAGGAATAGTCCT|ATGG|CTGGGTCAAATGGTATTTCTAGTTCTAGATCCT
                                                   >chr2:49442265-49442561 - E=4e-163 p
TGAGGAATCACCACACTGTCTTCCACAATGGTTGAACTAGTTTACACTCGCACCAGCAGTGTAAAAGCATTCCTATTTCTCCACATCCT
=0e+00
CTCCAGTACCTGTAGTTTCCTGACTTTTTAATGATCGCCATTCTAACTGGTATGAGATGGTATCTCATTGTGGTTTTGATTTGCATTTC

TCTGCTGACCAGTGATGATGAGGATTTTTTCACATGTCTGTTGGCTGCATAAATGTCTTCTTTTGAGAAGTGTCTGTTCAT|CCAC|TT
                                                                                      >
TGCCCACTTTTTAATGAGGTTGTTTGTTTTTCTCTTGTAAATTTGTC|AGATGCATAGTT|TGAAGAGTTCCTTAATCATTTTTGAAAC
chrX:124575077-124575126 + E=1e-11                          >chr10:20569625-20569900 +
AAAAATGTTAAGGGATTTTAAACATATGATTATTTTTAATTTTATGCCTTTTCAGTACTAAACACCCATTTCATTGCTGATTCCTGTCT
E=2e-153
AAGAAGCCATTCACGTCAGCATGGCGATAGAAAGAATGAAAAAACCCTGCTGAATCATACAGTAATTTTCTTTAAAGCACATAGTAGTT

ACATAAATATATATATATAAATATATTTTTGTTTATAACTAACACAAGGCAGGATCTTGTGACTCTAAG
```
